# Economic value of orthotic and prosthetic services among medicare beneficiaries: a claims-based retrospective cohort study, 2011–2014

**DOI:** 10.1186/s12984-018-0406-7

**Published:** 2018-09-05

**Authors:** Allen Dobson, Kennan Murray, Nikolay Manolov, Joan E. DaVanzo

**Affiliations:** Dobson DaVanzo & Associates, LLC, 450 Maple Avenue East, Suite 303, Vienna, VA 22180 USA

**Keywords:** Amputation, Cost-effectiveness, Limb-loss, Medicare, Orthoses, Prostheses, Rehabilitation

## Abstract

**Background:**

There are few studies of the economic value of orthotic and prosthetic services. A prior cohort study of orthotic and prosthetic Medicare beneficiaries based on Medicare Parts A and B claims from 2007 to 2010 concluded that patients who received timely orthotic or prosthetic care had comparable or lower total health care costs than a comparison group of untreated patients. This follow-up study reports on a parallel analysis based on Medicare claims from 2011 to 2014 and includes Part D in addition to Parts A and B services and expenditures. Its purpose is to validate earlier findings on the extent to which Medicare patients who received select orthotic and prosthetic services had less health care utilization, lower Medicare payments, and potentially fewer negative outcomes compared to matched patients not receiving these services.

**Methods:**

This is a retrospective cohort analysis of 78,707 matched pairs of Medicare beneficiaries with clinical need for orthotic and prosthetic services (*N* = 157,414) using 2011–2014 Medicare claims data. It uses propensity score matching techniques to control for observable selection bias. Economically, a cost-consequence evaluation over a four-year time horizon was performed.

**Results:**

Patients who received lower extremity orthotics had 18-month episode costs that were $1939 lower than comparable patients who did not receive orthotic treatment ($22,734 vs $24,673). Patients who received spinal orthotic treatment had 18-month episode costs that were $2094 lower than comparable non-treated patients ($23,560 vs $25,655). Study group beneficiaries receiving both types of orthotics had significantly lower Part D spending than those not receiving treatment (*p* < 0.05). Patients who received lower extremity prostheses had comparable 15-month episode payments to matched beneficiaries not receiving prostheses ($68,877 vs $68,893) despite the relatively high cost of the prosthesis.

**Conclusions:**

These results were consistent with those found in the prior study and suggest that orthotic and prosthetic services provide value to the Medicare program and to the patient.

## Background

Orthotic and lower extremity prosthetic devices and related clinical services are designed to provide patients with stability and mobility. While the literature contains considerable evidence of geographic variation in both major amputation rates and the use of orthotic and prosthetic (O&P) services [1–3], there are limited studies of the extent to which beneficiaries who receive O&P services experience a reduction in complications and/or costs with favorable outcomes [4].

While the variability in measures of quality and patient outcomes in research on O&P services can make comparisons difficult, studies have shown that the provision of O&P services led to measurable improvements in the quality of patient care and functional and psychosocial outcomes [5–7]. Beyond physical health, receipt of O&P services is associated with improved mental health status, in terms of social functioning, general health perception, and role limitation due to emotional problems [8]. The receipt of O&P services may also lead to societal gains including the return to work [9].

Additionally, O&P services can reduce health care spending via better patient outcomes, which in turn reduce other types of health care utilization [10, 11]. Long-term savings are thought to result when patients receive appropriate orthotic and prosthetic care. Without such care, individuals may live more sedentary lifestyles, which research has shown leads to secondary complications, such as diabetes and related comorbidities, as well as increases in health care utilization and spending [12]. Additionally, in some cases, the use of more sophisticated technology has been found to increase the quality of care and patient outcomes [13]. The beneficiary’s quality of life may very well be improved as well through increased mobility [14].

Our prior custom cohort study of orthotic and prosthetic Medicare beneficiaries that was based on Medicare claims experience over the 2007–2010 period found that the study group of patients who received timely orthotic or prosthetic care had lower total health care costs than a comparison group of untreated patients [10]. This study reports on a parallel analysis based on Medicare claims from 2011 to 2014 and includes Part D in addition to Parts A and B. Its primary objective is to validate earlier conclusions on the extent to which Medicare patients who received select orthotic and prosthetic services had less total health care utilization, lower Medicare payments, and/or fewer negative outcomes compared to matched patients not receiving these services. While the data are from Medicare only, the results of this study can inform the value proposition of orthotics and prosthetics for other payers.

## Methods

A retrospective cohort design of 78,707 one-to-one matched pairs of Medicare constituents (*N* = 157,414) was utilized. From an economic design type, a cost-consequence evaluation design was used with a total four-year time horizon. The payer’s perspective was selected for study to gain an understanding of value as it relates to orthotic and prosthetic provision under the Medicare program as a primary member of the reimbursement community. Study procedures were administered in accordance with the Declaration of Helsinki.

This study focuses on three types of O&P services – lower extremity orthoses, spinal orthoses, and lower extremity prostheses. The analytic methodology consisted of three key activities, including: 1) developing patient cohorts of orthotic and prosthetic users and matched comparison groups using a propensity score approach; 2) developing clinical episodes of care for each individual beneficiary; and 3) calculating descriptive statistics and analyzing the impact associated with each O&P service on Medicare episode utilization and payments.

### Developing patient cohorts

Analyses were conducted using Medicare claims from a custom database provided by the Centers for Medicare & Medicaid Services (CMS) (Data Use Agreement No. 28710). We requested a sample of beneficiaries with claims from 2011 to 2014 for patients with specified etiological diagnoses who received select lower extremity orthotic, spinal orthotic, or lower extremity prosthetic services. The etiological diagnosis related to the condition which ultimately led to the need for the lower extremity orthotic, spinal orthotic, or lower extremity prosthetic service (e.g., a functional diagnosis for a prosthetic device), not the diagnosis linked to the claims at the time of receipt of the service.^1^ These beneficiaries represented the study group population for each O&P service.

CMS identified the comparison (i.e., control) group population by matching beneficiaries to the patients who received orthotic and/or prosthetic devices (study group) based on the presence of an etiological diagnosis, gender, age, and state of residence. CMS provided up to five comparison group patients, who did not receive the select O&P services of interest, preliminarily matched to each study group patient.

The sampling methodology utilized by CMS to extract the custom cohorts allowed the analyses to reflect those Medicare beneficiaries who received an appropriate etiological diagnosis after January 1, 2011. Beneficiaries who died within three months of the etiological diagnosis were excluded from the cohorts. To be included in the study group, patients were required to have received specified orthotic or prosthetic services between January 1, 2012 and June 30, 2013. Beneficiaries in the prosthetic sample were required to have a relevant amputation documented in the claims during the study period. This sampling methodology ensured that the database included one year of claims prior to, and at least 18 months following, the receipt of the O&P service. Medicare health care claims across all care settings from 2011 to 2014 were obtained for the beneficiaries who met sampling specifications. Care settings included inpatient and outpatient hospitals, long-term care hospitals, skilled nursing facilities, inpatient rehabilitation facilities, home health agencies, hospice, physician/carrier visits, and durable medical equipment, prosthetics, orthotics, and supplies.

This database of study and comparison group beneficiaries served as the framework for the analytic sample selected using propensity score matching techniques. We used a one-to-one propensity score match across study and comparison group patients based on etiological diagnosis, comorbidities, patient sociodemographic characteristics (age, gender, race), and historical health care utilization. Additionally, because in the prosthetic analysis the clinical severity (and risk of imminent death) may have been a driver of whether or not the patient received a prosthesis, patients were also matched on the timing of death in relation to amputation, if applicable. As a result, mortality across the groups was excluded as a study outcome for the prosthetic analysis.

Propensity score matching techniques are widely used in observational studies when randomized controlled trials (RCTs) are not possible or are unethical or impractical to administer [15]. Literature suggests that applying these techniques to observational studies is an appropriate technique to remove observable selection bias among treatment and comparison groups and can result in findings that look like RCTs [16–19]. In addition, analyses based on administrative claims data are much less expensive than clinical trials.

Proper matching of the study and comparison group patients limited the number of episodes included in our study but helped to ensure that the study and comparison group patients were clinically and demographically similar [20]. Table 1 shows the number of study and comparison group patients included in each service group before and after matching. Propensity score matching resulted in 43,487 matched pairs of Medicare beneficiaries in the lower extremity orthotic model; 34,575 matched pairs in the spinal orthotic model; and 545 matched pairs of recent amputees in the prosthetic model. The number of orthotic patients in this current study is higher than in the 2007–2010 analysis, a designed increase in sample size resulting from the specifications of the custom cohort database. The relatively small number of beneficiaries included in the lower extremity prosthetic model was due to the requirement that amputation occur during the study window, which ensured the exclusion of long-term users who received replacement prosthetics during the study window, and also to the number of variables used in developing the propensity score match.

**Table 1:** Distribution of Pairs (Study Group and Comparison Group Matches)

	Lower extremity orthotic analysis		Spinal orthotic analysis		Lower extremity prosthetic analysis	
	Study group	Comparison group	Study group	Comparison group	Study group	Comparison group
Number of patients with O&P service and etiological diagnosis included in custom cohort	239,655	255,156	224,994	240,609	13,823	5959
Number of pairs after propensity score match	43,487	43,487	34,573	34,573	545	545
Percent of patients represented in the effective sample	18.1%	17.0%	15.4%	14.4%	3.9%	9.1%

Source: Dobson | DaVanzo analysis of custom cohort Standard Analytic Files (2011–2014) for Medicare beneficiaries who received O&P services from January 1, 2012 through June 30, 2013 (and matched comparisons), according to custom cohort database definition

### Developing episodes of care

Patient episodes were constructed to capture health care diagnoses, utilization, and expenditures prior to and after receipt of the orthotic or prosthetic device. Because actual costs were utilized in the analysis, and because at least one year of claims data prior to and after device provision was included, no additional discounting assumptions were incorporated. All patient episodes contained a pre-service window prior to the episode start, which allowed for the identification of comorbid conditions, patterns of institutional care, and other health care utilization used for risk-adjustment during the matching process. Episodes also contained a period of follow-up care, used to track trends in overall health care utilization, expenditures, and outcomes.

The episodes were structured similarly for the lower extremity and spinal orthotic analyses. For study group beneficiaries in these two service types, the post-service episode started upon receipt of the orthotic service, and the pre-service window comprised the 12 months prior to this date. The post**-**service period captured up to 18 months of Medicare claims after receiving the orthotic service. Because comparison group beneficiaries did not receive orthotic services, a proxy episode start date was established. To ensure the same post-service window for which health care utilization and expenditures were tracked and compared across cohorts, the length of time between etiological diagnosis and episode start, or “lag time,” for the comparison group was set to the average of the length of time for study group participants of similar age and gender. This lag time was added to the date of etiological diagnosis to create an episode start date for each comparison group beneficiary. Similar to the study group, the pre-service window comprised the 12 months prior to the episode start date, and the post-service window comprised the 18 months following the start date.

This episode structure was modified for the prosthetic analysis. In the 2007–2010 study, analysis using a temporal autocorrelation function indicated that the optimal length of the post-period for the prosthetic analysis was 12 months following the episode start, which was approximately three months after amputation. However, the Affordable Care Act (ACA) was implemented since our prior analysis, requiring modifications to this 2011–2014 study. The ACA had a considerable impact on hospital inpatient and outpatient mix, stay duration, and re-admission policies, among other factors. To address this, we used a 15-month episode period starting with the date of hospital discharge associated with amputation for the 2011–2014 lower extremity prosthetic population, as contrasted to the 3-month waiting period post-amputation and an immediately subsequent 12-month episode period we had used for the 2007–2010 study. Thus, both study and comparison groups had a pre-service window comprising the 12 months prior to this hospital discharge and a 15-month post-service window immediately following it.

### Calculating descriptive statistics and analyzing impact of orthotic/ prosthetic devices on overall patient Medicare expenditures

For each of the three analyses (lower extremity orthoses, spinal orthoses, and lower extremity prostheses), descriptive statistics were calculated for the study and comparison groups after the propensity score matching. The two groups were compared to each other based on the distribution of patient characteristics including but not limited to age, gender, race, and comorbidities. We then compared the total average episode Medicare payments of the study and comparison groups over the post-service period, as well as the distribution of payments by care settings, and a range of outcome measures, such as falls, hospitalizations, and days of rehabilitative/physical therapy.

## Results

### Demographic analysis

Table 2 presents the descriptive statistics of matched patients for each O&P service. Since the propensity score matching criteria included patient demographic characteristics and controlled for observable selection bias, the study and comparison group patients were highly similar within each O&P service type. No significant differences were found between the matched study and comparison groups for any variables used in the propensity score matching process, including age, gender, dual eligibility, and race, for any O&P service (*p* < 0.05).

**Table 2:** Descriptive Statistics across Matched Pairs (2011–2014)

	Lower extremity orthotic model		Spinal orthotic model		Lower extremity prosthetic model	
Demographic characteristic	Study group	Comparison group	Study group	Comparison group	Study group	Comparison group
Number of beneficiaries	43,487	43,487	34,575	34,575	545	545
Average age	68.6	68.7	67.2	67.2	65.9	65.9
Dual eligibility status	29.7%	29.7%	34.9%	34.9%	39.2%	39.2%
Gender: female	43.1%	43.1%	37.6%	37.6%	17.4%	17.4%
Race/Ethnicity: white	84.7%	84.7%	81.2%	81.2%	68.8%	68.8%
Race/Ethnicity: black or african american	8.3%	8.3%	11.8%	11.8%	24.8%	24.8%
Race/Ethnicity: hispanic	4.4%	4.4%	5.0%	4.4%	6.4%	6.4%

Differences were not significant at *α* = 0.05

Source: Dobson | DaVanzo analysis of custom cohort Standard Analytic Files (2011–2014) for Medicare beneficiaries who received O&P services from January 1, 2012 through June 30, 2013 (and matched comparisons), according to custom cohort database definition

Table 3 presents the ten most common etiological diagnoses for each type of O&P service, representing over 95% of beneficiaries in each service type. Because all matched pairs were required to have the same etiological diagnoses, the percentages are identical among the study and comparison groups, and Table 3 therefore presents the percent of matched pairs with each diagnosis. The most common etiological diagnosis for beneficiaries in the lower extremity orthotic analysis was other connective tissue disease, followed by spondylosis. These were also the top two diagnoses for beneficiaries in the spinal orthotic analysis, although the hierarchy was reversed. The most common diagnosis for beneficiaries in the lower extremity prosthetic analysis was diabetes mellitus with complications, followed by chronic ulcer of skin.

**Table 3:** Etiological Diagnoses across Matched Pairs (2011–2014)

Etiological diagnosis	Percent of matched pairs with diagnosis
Lower extremity orthoses	
Other connective tissue disease	32.4%
Spondylosis; intervertebral disc disorders; other back problems	17.9%
Other nervous system disorders	16.7%
Osteoarthritis	11.3%
Acute cerebrovascular disease	5.6%
Acquired foot deformities	3.8%
Fracture of lower limb	2.1%
Sprains and strains	2.1%
Multiple sclerosis	1.8%
Joint disorders and dislocations; trauma-related	1.5%
Spinal orthoses	
Spondylosis; intervertebral disc disorders; other back problems	40.1%
Other connective tissue disease	25.7%
Other nervous system disorders	15.6%
Osteoarthritis	7.7%
Other bone disease and musculoskeletal deformities	6.1%
Sprains and strains	2.0%
Other fractures	1.2%
Joint disorders and dislocations; trauma-related	0.7%
Other acquired deformities	0.4%
Other congenital anomalies	0.3%
Lower extremity prostheses	
Diabetes mellitus with complications	30.6%
Chronic ulcer of skin	18.0%
Peripheral and visceral atherosclerosis	17.8%
Other non-traumatic joint disorders	8.5%
Skin and subcutaneous tissue infections	7.9%
Other circulatory disease	4.9%
Complication of device; implant or graft	3.8%
Complications of surgical procedures or medical care	2.8%
Open wounds of extremities	2.7%
Infective arthritis and osteomyelitis	2.1%

Source: Dobson | DaVanzo analysis of custom cohort Standard Analytic Files (2011–2014) for Medicare beneficiaries who received O&P services from January 1, 2012 through June 30, 2013 (and matched comparisons), according to custom cohort database definition

### Outcomes analysis: lower extremity orthoses

Table 4 presents the health care utilization and payments by care setting for those who received lower extremity orthotic services (study group) compared to those who did not (comparison group). It presents the results of the updated 2011–2014 analysis as well as the results of the initial 2007–2010 analysis for comparison.

**Table 4:** Spending and Utilization for 18-Month Lower Extremity Orthotic Episode (2007–2010 and 2011–2014)

Care setting	2007–2010 analysis			2011–2014 analysis		
	*n* = 34,864 Matched pairs			*n* = 43,487 Matched pairs		
	Study	Comparison	Difference	Study	Comparison	Difference
Physician	$6482	$7171	-$688 ^*^	$5629	$6078	-$449 ^*^
DME	$2002	$966	$1036 ^*^	$763	$602	$162 ^*^
Acute Care Hospital / Other inpatient	$8392	$10,828	-$2436 ^*^	$5640	$6212	-$572 ^*^
Long Term Care Hospital	$366	$639	-$273 ^*^	$239	$294	-$55
Inpatient Rehabilitation Facility (IRF)	$1178	$924	$255 ^*^	$641	$378	$262 ^*^
Outpatient	$3552	$3752	-$199 ^*^	$2778	$3127	-$349 ^*^
Skilled Nursing Facility	$2415	$3180	-$765 ^*^	$1619	$1504	$115 ^*^
Home health	$2231	$1912	$320 ^*^	$1187	$908	$279 ^*^
Hospice	$388	$556	-$168 ^*^	$319	$607	-$288 ^*^
Total Part D Drug Spending	–	–	–	$3920	$4964	-$1044 ^*^
Total	$27,007	$29,927	-$2920 ^*^	$22,734	$24,673	-$1939 ^*^
Number of therapy visits	17.36	12.10	5.26 ^*^	12.53	4.93	7.60 ^*^
Number of fractures and falls	1.45	1.52	−0.07	0.38	0.48	−0.10 ^*^
Number of inpatient admissions	–	–	–	0.52	0.87	−0.35 ^*^
Length of stay for inpatient admissions (days)	–	–	–	2.64	4.77	−2.14 ^*^
Number of emergency room admissions	1.08	1.20	−0.12 ^*^	0.83	1.22	−0.39 ^*^
Number of IRF admissions	–	–	–	0.03	0.04	0.00 ^*^
Length of stay for IRF admissions (days)	0.72	0.52	0.20 ^*^	0.42	0.47	−0.05 ^*^
12-Month mortality rate	–	–	–	0.00	0.01	−0.01 ^*^

^*^ Difference is significant at *α* = 0.05

Source: Dobson | DaVanzo analysis of custom cohort Standard Analytic Files (2007–2010 and 2011–2014) for Medicare beneficiaries who received O&P services from January 1, 2008 through June 30, 2009 or January 1, 2012 through June 30, 2013 (and matched comparisons), according to custom cohort database definition

Across the 18-month episode, in this updated analysis the study group patients had a total Medicare payment of $22,734 compared to $24,673 for the comparison group, so the episode payment was $1939 lower for the study group (*p* < 0.05). A main cause for this difference was significantly fewer admissions to acute care hospitals, as the study group patients were admitted 0.52 times during the episode, compared to 0.87 times for the comparison group (*p* < 0.05). This lower rate of utilization lowered the total episode payments by $572 for patients receiving orthoses.

In addition, similar to the 2007–2010 analysis, we again found that the lower extremity orthotic study group had significantly lower payments to physicians and outpatient hospitals. Study group beneficiaries also had lower overall Part D drug spending, a significant difference of $1044 (*p* < 0.05).

Despite having lower total episode payments, beneficiaries receiving the lower extremity orthoses demonstrated significantly higher expenditures in most post-acute care settings, including inpatient rehabilitation facilities ($641 vs $378), skilled nursing facilities ($1619 vs $1504), and home health ($1187 vs $908) (*p* < 0.05). These results are similar to those of the 2007–2010 analysis, with the exception of skilled nursing facilities. In the earlier analysis, expenditures in this care setting were $765 less than the comparison group across the 18-month episode. In addition, patients who received lower extremity orthoses received significantly more outpatient therapy than those who did not receive the orthotic (12.53 vs 4.93 visits, *p* < 0.05). As shown in Table 4, analysis of other outcomes revealed that study group patients experienced significantly fewer falls and fractures (0.38 compared to 0.48, *p* < 0.05) and significantly fewer emergency room (ER) admissions (0.83 vs 1.22, *p* < 0.05).

**Fig. 1:**
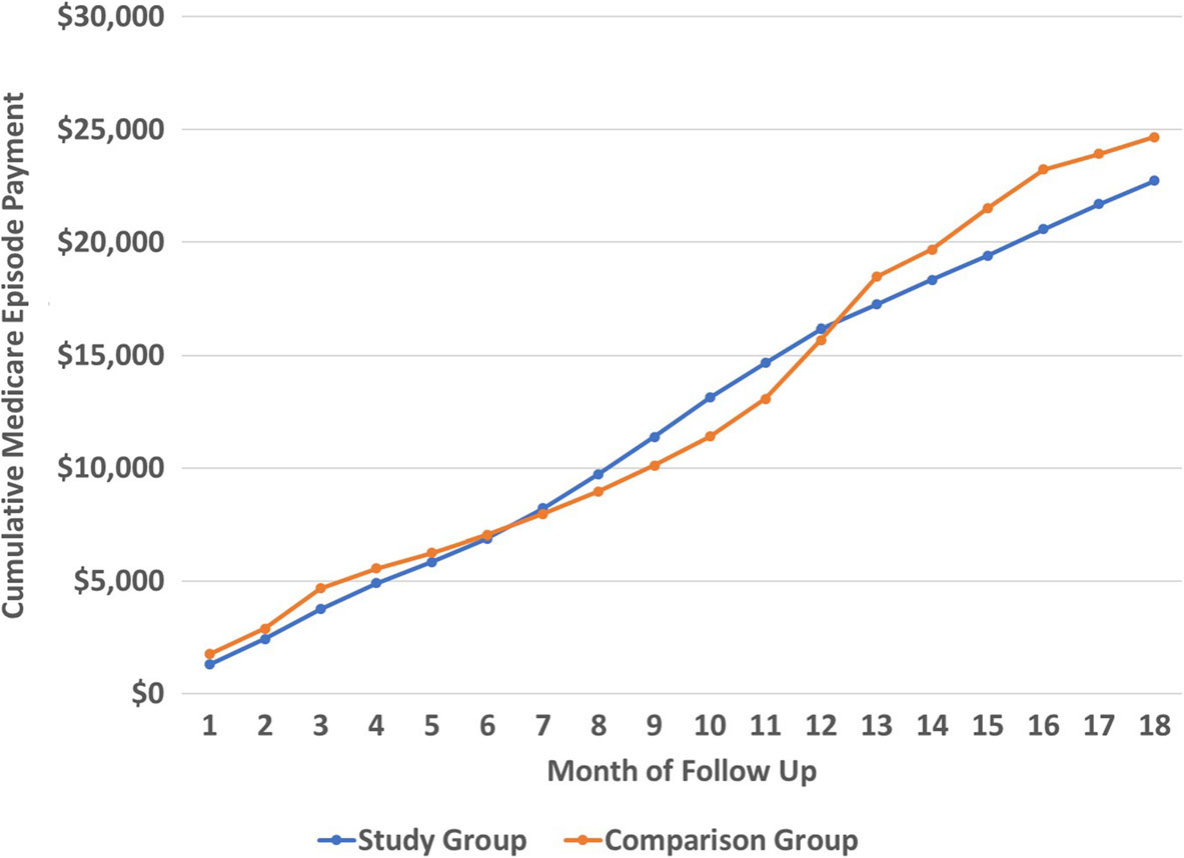
Cumulative Lower Extremity Orthotic Episode Payment by Cohort

Figure 1 presents the cumulative episode payment for those who received the lower extremity orthoses compared to those who did not by episode month. Despite a period of higher spending in Months 7 to 12, the study group patients had lower Medicare episode payments than the comparison group. Thus, over the entire 18-month episode the cost of the orthotic was fully amortized through reduced utilization in other settings. These findings are consistent with those of the 2007–2010 analysis.

### Outcomes analysis: spinal orthoses

Table 5 presents the health care utilization and payments by care setting for those patients who received spinal orthoses (study group) compared to those who did not (comparison group). Across the 18-month episode, the study group patients had significantly lower total episode payments across all care settings ($23,560 vs $25,655, *p* < 0.05). This result is different than that found in the 2007–2010 analysis, which found a nonsignificant difference in total episode spending between the study and comparison groups.

**Table 5:** Spending and Utilization for 18-Month Spinal Orthotic Episode (2007–2010 and 2011–2014)

	2007–2010 analysis			2011–2014 analysis update		
	*n* = 6247 Matched pairs			*n* = 34,575 Matched pairs		
Care setting	Study	Comparison	Difference	Study	Comparison	Difference
Physician	$7907	$7439	$468*	$6291	$6570	-$279*
DME	$2605	$1288	$1317*	$722	$621	$101*
Acute Care Hospital / Other inpatient	$11,373	$11,830	-$457	$5913	$6294	-$381*
Long Term Care Hospital	$517	$837	-$320**	$190	$269	-$79*
Inpatient Rehabilitation Facility (IRF)	$990	$1188	-$198**	$433	$341	$92*
Outpatient	$3786	$4120	-$334	$2734	$3294	-$559*
Skilled Nursing Facility	$2188	$3175	-$987*	$1234	$1281	-$47*
Home Health	$2802	$2388	$414*	$1100	$901	$199*
Hospice	$431	$426	$5**	$234	$534	-$300*
Total Part D Drug Spending	–	–	–	$4709	$5550	-$840*
Total	$32,598	$32,691	-$93	$23,560	$25,655	-$2094*
Average number of therapy visits	14.95	12.91	2.04	6.14	2.06	4.08*
Average number of fractures and falls	2.05	1.56	0.50*	0.32	0.32	0.00
Average number of inpatient admissions	–	–	–	0.40	0.68	−0.28*
Length of Stay for inpatient admissions (days)	–	–	–	1.84	3.53	−1.69*
Average number of emergency room admissions	1.35	1.32	0.03	0.81	1.03	−0.23*
Average number of IRF Admissions	–	–	–	0.02	0.03	−0.01*
Length of Stay for IRF Admissions (days)	0.62	0.68	−0.06	0.24	0.32	−0.07*
12-Month Mortality Rate	–	–	–	0.00	0.01	−0.01*

* Difference is significant at *α* = 0.05

** The difference in spending between the study and comparison groups for IRF, LTCH, Other Inpatient and Hospice settings combined was significant at *α* = 0.05

Source: Dobson | DaVanzo analysis of custom cohort Standard Analytic Files (2007–2010 and 2011–2014) for Medicare beneficiaries who received O&P services from January 1, 2008 through June 30, 2009 or January 1, 2012 through June 30, 2013 (and matched comparisons), according to custom cohort database definition

In this updated analysis, a major contributor to the difference in total episode payments between the study and comparison groups was significantly lower payments for Part D drugs in the study group ($840 lower among Part D users only, *p* < 0.05). Study group patients had higher payments for DME services, inpatient rehabilitation facilities, and home health, but lower payments to acute care hospitals, long-term care hospitals and physician offices (*p* < 0.05). This is somewhat different than our earlier analysis, which found higher payments to physician offices and lower payments to inpatient rehabilitation facilities.

Despite higher payments for inpatient rehabilitation care in the study group, the average length of stay in inpatient rehabilitation facilities was significantly lower in this group (0.24 vs 0.32, *p* < 0.05). These patients appear more likely to return home faster and to receive follow up care in the home, as evidenced by higher payments to home health among the study group ($1100 vs $901, *p* < 0.05).

Study group patients who received spinal orthoses experienced the same number of fractures and falls compared to those who did not receive the orthoses, but a significantly lower number of emergency room admissions (0.81 admissions for the study group compared to 1.03 for the comparison group, *p* < 0.05).

**Fig. 2:**
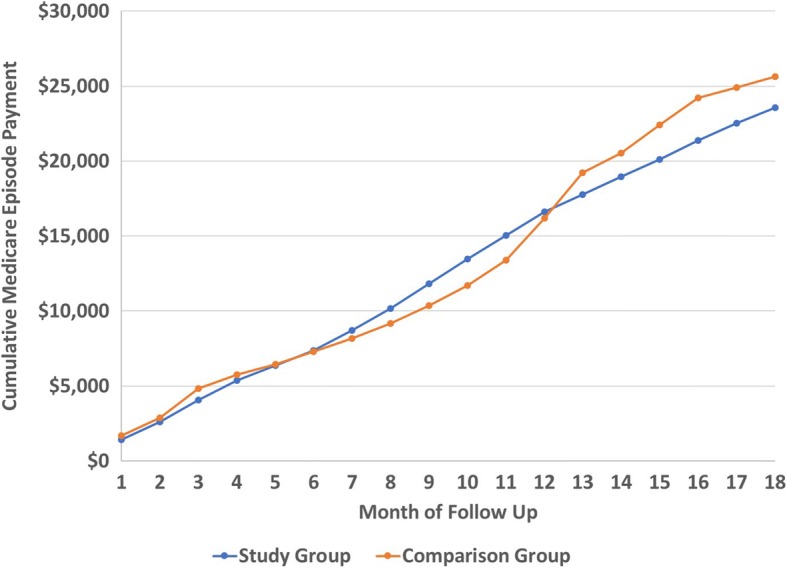
Cumulative Spinal Orthotic Episode Payment by Cohort

Figure 2 presents the cumulative episode payment for those who received spinal orthoses compared to those who did not by episode month. Similar to the lower extremity orthotic analysis, this chart indicates that, despite a period of additional cost for the study group between months 7 to 12, the cost of the orthotic was fully amortized over the episode.

### Outcomes analysis: lower extremity prostheses

Table 6 presents the health care payments by care setting for those who received lower extremity prostheses compared to those who did not. As discussed in the methodology, the results for lower extremity prostheses were compared across approximately 15 months post-service.

**Table 6:** Spending and Utilization for 18-Month Lower Extremity Prosthetic Episode (2007–2010 and 2011–2014)

Care setting	2007–2010 analysis			2011–2014 analysis update		
	*n* = 428 Matched pairs			*n* = 545 Matched pairs		
	Study	Comparison	Difference	Study	Comparison	Difference
Physician	$7792	$11,883	-$4092*	$8270	$9920	-$1649
DME	$18,653	$2537	$16,116*	$15,323	$5018	$10,305*
Prosthetics Only: L5000 - L5999	–	–	–	$9694	$1782	$7912*
Acute Care Hospital / Other Inpatient	$18,080	$28,276	-$10196*	$15,529	$19,851	-$4321*
Long Term Care Hospital	$1408	$4102	-$2694**	$1445	$4017	-$2571*
Inpatient Rehabilitation Facility (IRF)	$2603	$2000	$603**	$3476	$3415	$61
Outpatient	$9373	$7291	$2082*	$8601	$8649	-$49
Skilled Nursing Facility	$8386	$8821	-$435	$5783	$6630	-$847
Home Health	$6181	$5692	$489	$5049	$4764	$285
Hospice	$715	$1572	-$857**	$104	$825	-$721*
Total Part D Drug Spending	–	–	–	$5297	$5806	-$508
Total	$73,191	$72,175	$1015	$68,877	$68,893	-$16
Average number of therapy visits	56.10	28.90	27.20*	26.86	17.97	8.89*
Average number of fractures and falls	0.90	0.72	0.18	0.46	0.41	0.05
Average number of inpatient admissions	1.18	1.51	−0.33	1.23	1.54	−0.31*
Length of stay for inpatient admissions (days)	–	–	–	7.53	11.44	−3.91*
Average number of emergency room admissions	1.55	2.10	−0.55*	2.14	2.03	0.11
Average number of IRF admissions	–	–	–	0.17	0.14	0.02
Length of stay for IRF admissions (days)	1.61	1.19	0.42	2.16	2.10	0.07

* Difference is significant at *α* = 0.05

** The difference in spending between the study and comparison groups for IRF, LTCH, Other Inpatient and Hospice settings combined was significant at *α* = 0.05

Source: Dobson | DaVanzo analysis of custom cohort Standard Analytic Files (2007–2010 and 2011–2014) for Medicare beneficiaries who received O&P services from January 1, 2008 through June 30, 2009 or January 1, 2012 through June 30, 2013 (and matched comparisons), according to custom cohort database definition

Across the 15-month episode, the study group patients had total Medicare payments that were slightly, but not significantly, lower than the comparison group ($68,877 for the study group compared to $68,893 for the comparison group). About 14% of the total episode payment for the study group patients is attributed to the prosthesis ($9694 of the total episode payment of $68,877). The prosthetic device represents an additional cost that was fully amortized within 15 months due to a reduction of care in other settings. This stands in contrast to the 2007–2010 analysis, which found higher total episode payments of $1015 among the study group.

The largest difference in payments between the study and comparison groups was for acute care hospitals. The study group patients had a significantly lower rate of hospitalization than the comparison group patients (1.23 admissions for the study group compared to 1.54 admissions for the comparison group, *p* < 0.05), resulting in lower episode Medicare payments for acute care hospitalizations ($15,529 for the study group compared to $19,851 for the comparison group, *p* < 0.05). These results are similar to those found in the 2007–2010 analysis.

Study group patients had significantly lower expenditures for facility-based long-term care and in-home hospice services than the comparison group patients (*p* < 0.05), but spending differences were not significantly different in other care settings. Expenditures were nominally lower among study group participants in physician offices, hospital outpatient departments, and skilled nursing facilities, but nominally higher among study group participants for inpatient rehabilitation facilities and home health. In addition, expenditures were lower for Part D drugs among the study group, although this difference was not significant.

Patients need to be trained and receive extensive therapy to properly use a prosthetic device, and study group patients had considerably higher utilization of outpatient therapy (26.86 visits vs 17.97 visits, *p* < 0.05). The number of fractures and falls and emergency room admissions were not significantly different between the study and comparison groups.

**Fig. 3:**
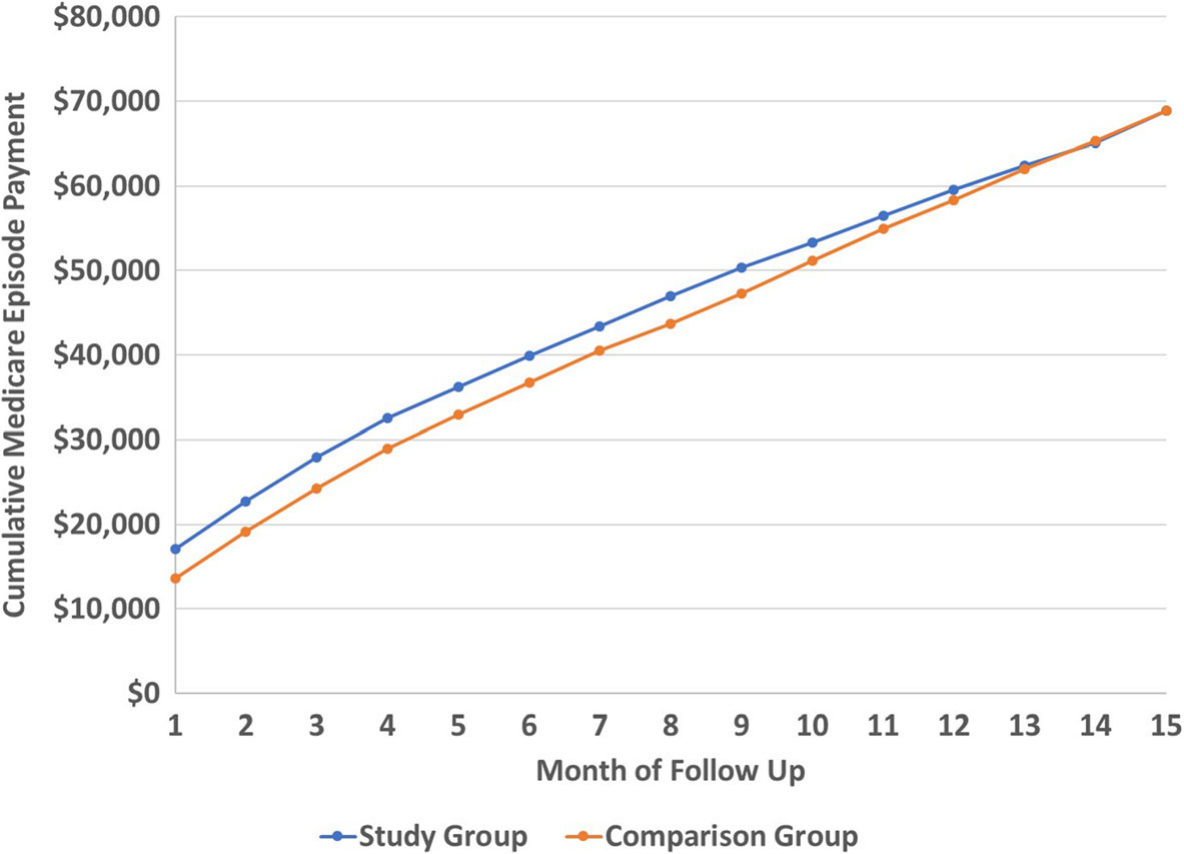
Cumulative Lower Extremity Prosthetic Episode Payment by Cohort

Figure 3 presents the cumulative episode payment for the study and comparison group by episode month. This chart indicates that the cost of the prosthetic was slowly amortized over time; by the end of Month 15, the cumulative Medicare episode payment for the study group was similar to that of the comparison group, indicating that the cost of the prosthetic was fully amortized.

## Discussion

The literature indicates that the receipt of orthotic and prosthetic services could increase a patient’s mobility, ultimately reducing their health care utilization and increasing their quality of life. Based on this possibility, this study investigated the economic impact and value of lower extremity orthoses, spinal orthoses, and lower extremity prostheses. Propensity score matching techniques allowed for the comparison of clinically and demographically similar patients who received these services to those who did not, and thus for a determination of the economic impact of these services on the Medicare population. Because this study is based on Medicare claims data, it excludes some other sources of economic value and outcomes, such as the ability for patients with prostheses to return to work or become more independent from social services. These are sources of economic impact from the societal and consumer’s perspective, although they are not generally relevant to the largely nonworking Medicare population and were outside the scope of the current analysis.

Results indicated that over an 18-month period, patients who received lower extremity orthotics or spinal orthotics had reduced Medicare payments. Savings were in the range of $2000 for both types of orthotic services, or approximately 8% of total Medicare health costs in the follow-up period. Beneficiaries who received lower extremity prostheses had similar total episode payments over 15 months, despite the higher cost of the prosthetic device, due to lower expenditures in other care settings.

Within the lower extremity orthotics analysis, these results demonstrated lower payments to physicians, outpatient hospitals, and for Part D drugs. This may suggest overall lower morbidity or comorbidity in patients who receive the orthotic service. In addition, higher utilization of post-acute care may be an important reason why acute care hospital admissions and expenditures are significantly lower in the study group. That is, the higher use of post-acute care may eliminate the need for additional or subsequent admission to acute care hospitals, ultimately lowering total episode cost. The increased rate of outpatient therapy seen in the study group is consistent with Medicare’s emphasis on restorative care for beneficiaries, when possible. It may be related to the lower rate of negative outcomes for patients who received O&P services, including fewer fractures and falls and emergency room visits. The results of this analysis suggest that with the receipt of the lower extremity orthotic, study group patients could withstand more intensive therapy that led to in increased standing stability, resulting in fewer emergency room admissions, hospitalizations, and lower Medicare payments.

In the spinal orthotic model, the lower payments for Part D drugs seen among study group beneficiaries could indicate lower prevalence of comorbid conditions and generally better health status among beneficiaries receiving spinal orthoses, compared to those who do not. Differences between this updated analysis and the previous one suggest that there may have been a different standard of care for patients receiving spinal orthotics in 2011–2014 than there was in 2007–2010. This updated analysis found higher payments for rehabilitation facilities among study group participants, which could indicate a shift toward more intensive facility-based rehabilitative care for beneficiaries receiving orthoses.

This analysis of lower extremity prosthetic services demonstrated that the cost of the prosthetic device and clinical prosthetic care was amortized within the 15-month follow-up period, offset by higher total costs for the untreated comparison group patients. Comparative efficacy trials and systematic reviews of components have found similar value concluding that some prosthetic components may be initially costlier but are ultimately worth funding due to lower fall risk, less work missed and improved quality of life [4, 14, 21]. In this study, through a reduction in acute care hospitalizations, physician visits, and facility-based care, patients experienced improved quality of life at a comparable Medicare episode payment.

Study and comparison group beneficiaries in this lower extremity prosthetic analysis had roughly a comparable number of fractures and falls, as well as comparable emergency room admission among lower extremity prosthetic users, compared to those who did not receive the service. Part of the savings due to reduced facility-based care was offset by more extensive physical therapy and rehabilitation presumably to teach patients how to properly use their prostheses, as amputees must learn balance and mobility with their new device. Additionally, the high use of therapy among beneficiaries in the study group may be associated with increased ambulation, which suggests that the study group patients with prostheses were less homebound than the comparison group. This increased level of independence among beneficiaries receiving prostheses may explain the similarity in the rate of falls and fractures and emergency room admissions among the study and comparison groups.

Much has changed in health care, and in orthotic and prosthetic care, since 2010. Despite research that suggests that O&P services can prevent falls, reduce downstream clinical manifestations such as the development of diabetes, and lead to long-term savings in health care spending, patients can face significant barriers to access. Varying cost pressures caused Medicare prosthetic payments to decline by 6% between 2010 and 2014, and Medicare beneficiary access to more advanced prosthetics declined even more steeply, by approximately 36% over that same period [22]. In 2015, Medicare contractors proposed a new Local Coverage Determination (LCD) which would have further restricted access to more advanced devices, asserting, for example, that any Medicare beneficiary who had received a walker, wheel chair, crutches or cane would be automatically excluded from eligibility for more advanced devices. This proposed LCD prompted such controversy that the entire matter was referred to study, which has continued for nearly two years without any published conclusions. In the interim, the RAND Corporation has issued a new report underscoring the economic value of advanced technologies for amputees [23].

Our study suggest that lower extremity orthoses, spinal orthoses and lower extremity prostheses have the potential to increase quality of life and reduce facility-based care for applicable Medicare beneficiaries. Similarly, these results suggest that orthotic and prosthetic services provide value to the Medicare program, as well as to the patient. In orthotics, there is a clear savings margin for the treated study group patients. In prosthetics, the cost of the services, including the higher initial cost of the prosthesis itself, is completely amortized through reduced acute care hospitalizations and facility-based care. One clinical example of this is the situation where microprocessor knees have been shown to improve patient safety in patients with transfemoral amputation by reducing stumble and fall events [11].

### Limitations

One limitation of the methodology was reliance on administrative data as opposed to clinical data recorded in the medical records. While the dataset included all fee-for-service health care utilization and payments, more detailed clinical indicators, such as functional status, were not available from the administrative data. Propensity score matching relied on all recorded patient demographic and clinical characteristics in an attempt to control for observable selection bias among those who received orthotic/prosthetic services compared to those who did not. More medical information could perhaps improve the selection of matched pairs.

Another limitation of the claims data was the lack of Medicare Advantage discharges and Medicaid long term care-related expenses for dually eligible patients. The relationship of the Medicare to Medicaid payment systems is problematic for analyses that involve episodes of care, as the exclusion of Medicaid claims for dually eligible patients prohibits identification of patients who receive care in long-term care facilities as compared to the community. With additional data, reduction in long-term care facility use may have been determined to be another important outcome variable for the study group.

## Conclusion

The results of this study generally echo those of the prior study, with some fluctuation in the cost difference between the study and comparison groups in specific subcategories of expenditures. Study group patients receiving lower extremity and spinal orthoses had significantly lower total episode spending than did the non-treated beneficiaries in the comparison group, despite having more therapy visits. Study group patients receiving lower extremity prostheses had average Medicare payments across all care settings that were slightly lower than the comparison group and the prosthetic cost was fully amortized within 15 months due to a reduction of care in other settings. Among other identified benefits to prosthetic use, prosthesis users had a significantly lower hospitalization rate than comparison group patients further resulting in lower Medicare payments for acute care hospitalizations. Across all analyses, the results cumulatively suggest that orthotic and prosthetic services provide value to the Medicare program, and potentially to other payers, as well as to the patient.

## Abbreviations

ACA: Affordable Care Act
CMS: Centers for Medicare & Medicaid Services
IRF: Inpatient Rehabilitation Facility
LCD: Local Coverage Determination
O&P: Orthotics and prosthetics
RCT: Randomized controlled trial

## Footnotes

1 Codes used to identify the etiological diagnoses of interest for the orthotic and prosthetic models are included in a separate technical methodology available from the authors.
